# Interleukin-17A Differentially Induces Inflammatory and Metabolic Gene Expression in the Adipose Tissues of Lean and Obese Mice

**DOI:** 10.3390/ijms17040522

**Published:** 2016-04-07

**Authors:** Yine Qu, Qiuyang Zhang, Siqi Ma, Sen Liu, Zhiquan Chen, Zhongfu Mo, Zongbing You

**Affiliations:** 1 Department of Structural and Cellular Biology, Tulane University, New Orleans, LA 70112, USA; linlin0852@sina.com (Y.Q.); qzhang3@tulane.edu (Q.Z); sliu1@tulane.edu (S.L.); czq680302@163.com (Z.C.); mozhongfu1988@gmail.com (Z.M.); 2 Department of Histology and Embryology, School of Basic Medicine of North China University of Science and Technology, Tangshan 063000, China; jckyx@sina.com; 3 Department of Thoracic Surgery, Affiliated Hospital of North China University of Science and Technology, Tangshan 063000, China; 4 Department of Obstetrics and Gynecology, Shijiazhuang Maternal and Child Health Care Hospital, Shijiazhuang 050000, China; 5 Department of Orthopaedic Surgery, Tulane University, New Orleans, LA 70112, USA; 6 Tulane Cancer Center and Louisiana Cancer Research Consortium, Tulane University, New Orleans, LA 70112, USA; 7 Tulane Center for Stem Cell Research and Regenerative Medicine, Tulane University, New Orleans, LA 70112, USA; 8 Tulane Center for Aging, Tulane University, New Orleans, LA 70112, USA

**Keywords:** interleukin-17A, inflammation, metabolism, obesity, gene expression

## Abstract

The functions of interleukin-17A (IL-17A) in adipose tissues and adipocytes have not been well understood. In the present study, male mice were fed with a regular diet (*n* = 6, lean mice) or a high-fat diet (*n* = 6, obese mice) for 30 weeks. Subcutaneous adipose tissue (SAT) and visceral adipose tissue (VAT) were analyzed for IL-17A levels. SAT and VAT were treated with IL-17A and analyzed for inflammatory and metabolic gene expression. Mouse 3T3-L1 pre-adipocytes were differentiated into adipocytes, followed with IL-17A treatment and analysis for inflammatory and metabolic gene expression. We found that IL-17A levels were higher in obese SAT than lean SAT; the basal expression of inflammatory and metabolic genes was different between SAT and VAT and between lean and obese adipose tissues. IL-17A differentially induced expression of inflammatory and metabolic genes, such as tumor necrosis factor α, *Il-6*, *Il-1*β, *leptin*, and glucose transporter 4, in adipose tissues of lean and obese mice. IL-17A also differentially induced expression of inflammatory and metabolic genes in pre-adipocytes and adipocytes, and IL-17A selectively activated signaling pathways in adipose tissues and adipocytes. These findings suggest that IL-17A differentially induces inflammatory and metabolic gene expression in the adipose tissues of lean and obese mice.

## 1. Introduction

Obesity is defined as having a body mass index (BMI) ≥30 kg/m^2^ in the adults (>19 years old). Approximately 35% of adult Americans are obese [1]. Obesity is associated with type 2 diabetes mellitus, cardiovascular disease, stroke, and some types of cancer [2]. The World Health Organization predicts a 75% surge of cancer occurrence worldwide in the next 20 years and reducing the obesity rate may prevent many cancers [3]. Cancer-specific mortality is significantly increased in obese men and women with many common cancer types, such as cancers of the liver, pancreas, stomach, esophagus, colon and rectum, gallbladder, kidney, prostate, breast, uterus, cervix, and ovary, as well as multiple myeloma and non-Hodgkin’s lymphoma [4].

It is well known that obesity is a chronic inflammatory status with increased serum levels of tumor necrosis factor-α (TNF-α), interleukin-6 (IL-6), and IL-1β [5,6,7]. The nucleotide-binding domain, leucine-rich-containing family, pyrin domain-containing-3 (abbreviated as NLRP3) inflammasome senses obesity-associated danger signals, leading to caspase-1 activation and subsequent secretion of IL-1β and IL-18 [6]. Infiltration of innate and adaptive immune cells into adipose tissue is linked to metabolic dysfunctions [2]. In obesity, adipose tissue expansion is accompanied with an acute infiltration of neutrophils, which are followed by B and T cells and then by macrophages. The adipose tissue contains increased number of M1 (classically-activated) macrophages, neutrophils, mast cells, CD8+ T cells, CD4+ Th1 cells, natural killer cells, and B cells, which are pro-inflammatory [8].

Several studies have found that serum levels of IL-17 (also called IL-17A) are increased in obese mice [9,10] and humans [11]. IL-17A is a key cytokine that plays important roles in many inflammatory and autoimmune diseases as well as some cancers [12,13]. IL-17A can induce expression of TNF-α, IL-6, and IL-1β cytokines [14], which are found with increased levels in obesity. It has been demonstrated that IL-17A can inhibit differentiation of pre-adipocytes into adipocytes through suppressing expression of pro-adipogenic peroxisome proliferator-activated receptor γ (PPARγ), CCAAT/enhancer-binding protein α (C/EBPα), and Kruppel-like family 15 (KLF15), but enhancing expression of anti-adipogenic KLF2 and KLF3 [15]. IL-17A receptor C (IL-17RC) expression has been found in human mature adipocytes [16]. However, little information is known about IL-17A’s effects on mature adipocytes or adipose tissue. The purpose of the present study was to assess IL-17A’s functions in adipose tissue. We found that IL-17A differentially induced inflammatory and metabolic gene expression in the adipose tissues of lean and obese mice.

## 2. Results

### 2.1. IL-17A Levels Are Increased in Subcutaneous Adipose Tissue (SAT) of Obese Mice

**Figure 1:**
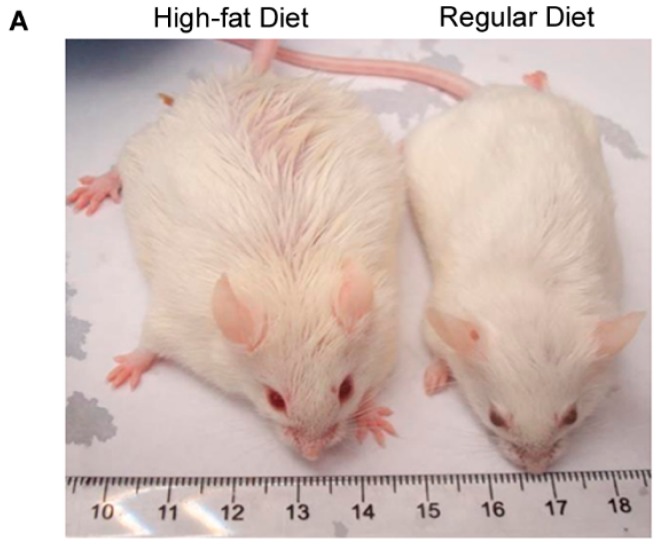

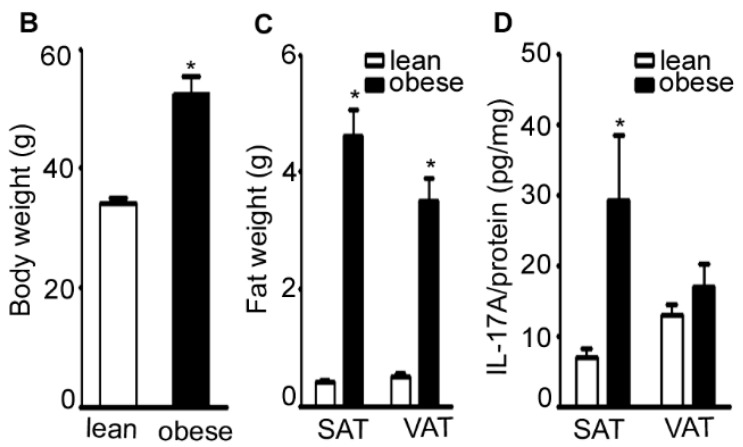
Interleukin-17A (IL-17A) levels are increased in SAT of obese mice. (**A**) Representative pictures of obese mice (*n* = 6) fed with a high-fat diet and lean mice (*n* = 6) fed with a regular diet at age of 30 weeks; (**B**) animal body weight mean ± SEM (error bars), *n* = 6 per group, * *p* < 0.001; (**C**) adipose tissue weight mean ± SEM (error bars), *n* = 6 per group, * *p* < 0.001; and (**D**) IL-17A levels (pg/mg tissue protein) in adipose tissues, mean ± SEM (error bars), *n* = 6 per group, * *p* < 0.05. SAT: Subcutaneous adipose tissue; VAT: visceral adipose tissue.

Mice fed with a high-fat diet became obese at age of 30 weeks (Figure 1A) with a body weight approximately 47% heavier than mice (named as lean mice) fed with a regular diet (Figure 1B, *p* < 0.001). The weight of subcutaneous adipose tissue (SAT) and visceral adipose tissue (VAT) were also significantly heavier in obese mice than lean mice (Figure 1C, *p* < 0.001). IL-17A levels were significantly higher in obese SAT than lean SAT (*p* < 0.05), while IL-17A levels in obese VAT were slightly, but insignificantly, increased compared to lean VAT (Figure 1D).

### 2.2. IL-17A Differentially Induces Inflammatory Gene Expression in the Adipose Tissues of Lean and Obese Mice

**Figure 2:**
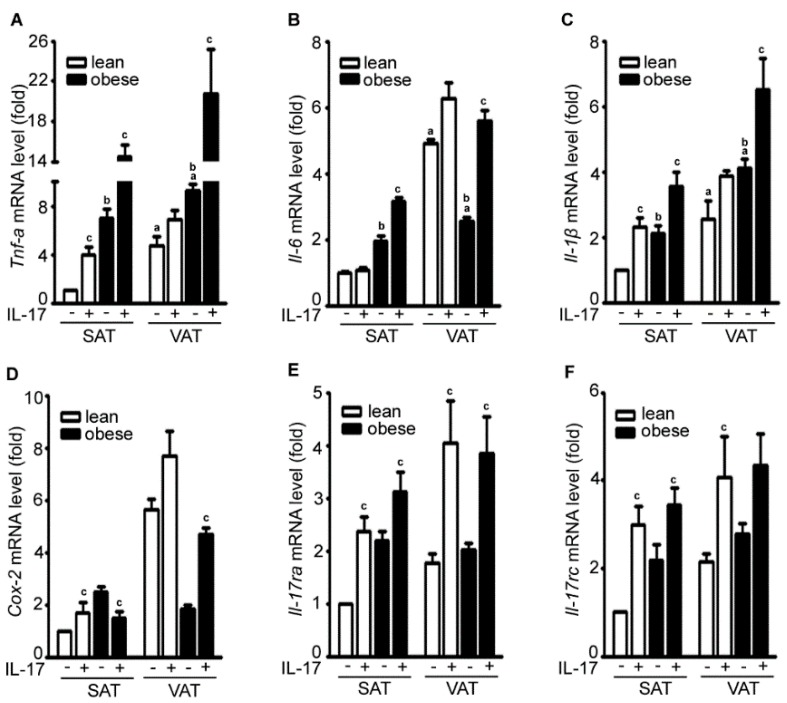

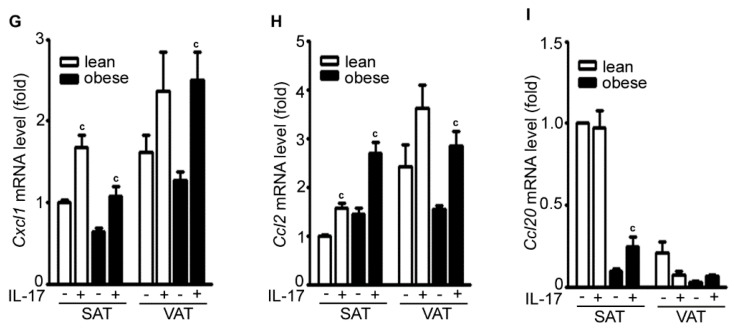
IL-17A differentially induces inflammatory gene expression in the adipose tissues of lean and obese mice. (**A**–**I**) Adipose tissues were cultured in serum-free Dulbecco’s Modified Eagle Medium (DMEM) and treated without (−) or with (+) 20 ng/mL IL-17A for 2 h; mRNA expression of each gene was analyzed with qRT-PCR; SAT without IL-17A treatment served as the basal level for comparison; data represent mean ± SEM (error bars), *n* = 6 per group; ^a^ *p* < 0.05, compared to the untreated SAT group; ^b^ *p* < 0.05, compared to the lean mice; ^c^ *p* < 0.05, comparison between the untreated and IL-17A-treated groups.

Using qRT-PCR to assess mRNA expression, we found that the basal levels (*i.e.*, the control group without IL-17A treatment) of *Tnf-*α, *Il-6*, and *Il-1*β were higher in VAT than SAT in both lean and obese mice (Figure 2A–C). The basal levels of *Tnf-*α, *Il-6*, and *Il-1*β were higher in the obese adipose tissues than lean adipose tissues except *Il-6* in VAT (Figure 2A–C). IL-17A treatment increased expression of *Tnf-*α, *Il-6*, and *Il-1*β in SAT and VAT with a higher amplitude in obese adipose tissues than lean adipose tissues (Figure 2A–C). IL-17A treatment increased cyclooxygenase-2 (*Cox-2*) expression in lean SAT, lean VAT, and obese VAT, but slightly decreased *Cox-2* expression in obese SAT (Figure 2D). IL-17A treatment also increased expression of its own receptors *IL-17ra* and *IL-17rc* in SAT and VAT of both lean and obese mice (Figure 2E,F). IL-17A treatment induced expression of C–X–C motif ligand 1 (*Cxcl1*) and C–C motif ligand 2 (*Ccl2*) in SAT and VAT of both lean and obese mice (Figure 2G,H). However, IL-17A treatment did not increase *Ccl20* expression except a slight increase in obese SAT (Figure 2I).

### 2.3. IL-17A Differentially Induces Metabolic Gene Expression in the Adipose Tissues of Lean and Obese Mice

**Figure 3:**
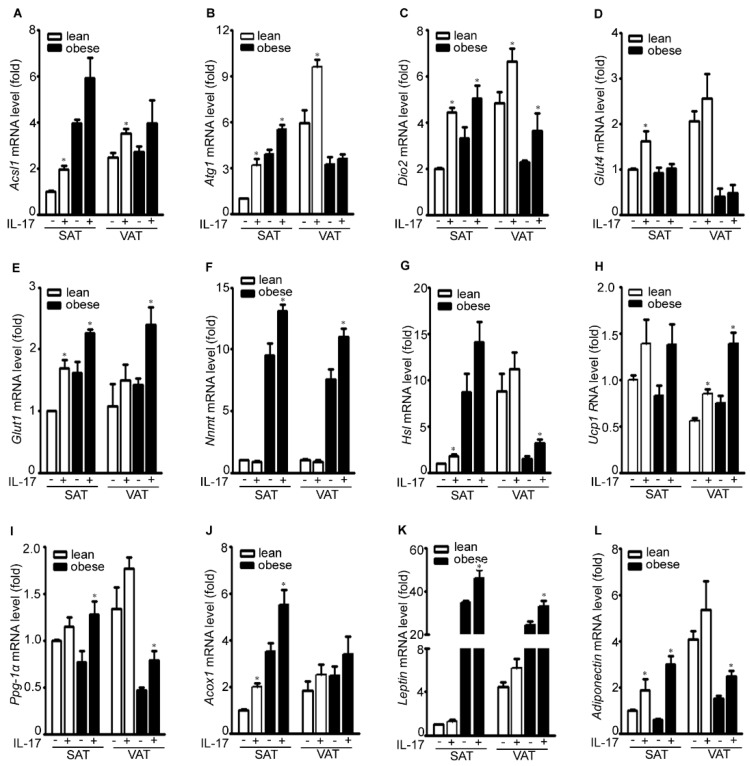
IL-17A differentially induces metabolic gene expression in the adipose tissues of lean and obese mice. (**A**–**L**) Adipose tissues were cultured in serum-free DMEM and treated without (−) or with (+) 20 ng/mL IL-17A for 2 h; mRNA expression of each gene was analyzed with qRT-PCR; SAT without IL-17A treatment served as the basal level for comparison; data represent mean ± SEM (error bars), *n* = 6 per group; * *p* < 0.05, comparison between the untreated and IL-17A-treated groups.

IL-17A treatment increased expression of acyl-CoA synthetase long-chain family member 1 (*Acsl1*) in SAT and VAT of lean and obese mice (Figure 3A). The *Acsl1* gene encodes an enzyme to convert free long-chain fatty acids into fatty acyl-CoA esters, thereby playing a key role in lipid biosynthesis and fatty acid degradation. IL-17A treatment increased expression of autophagy related 1 (*Atg1*) in lean SAT, obese SAT, and lean VAT, but not in obese VAT (Figure 3B). *Atg1* gene-encoded protein forms a complex with other Atg proteins to sense nutrient availability. IL-17A treatment increased expression of deiodinase, iodothyronine, type II (*Dio2*) in SAT and VAT of lean and obese mice (Figure 3C). *Dio2* gene-encoded protein is responsible for deiodination of T4 (3,5,3,5-tetraiodothyronine) into T3 (3,5,3-triiodothyronine), an important hormone in metabolism. It has been reported that diet-induced obesity is mediated by the JNK/DIO2 signal transduction pathway [17]. IL-17A treatment increased expression of glucose transporter 4 (*Glut4*) in SAT and VAT of lean mice, but not of obese mice (Figure 3D). In contrast, IL-17A induced *Glut1* expression in SAT and VAT of both lean and obese mice (Figure 3E). Glut proteins facilitate transport of glucose across the plasma membranes of mammalian cells. On the other hand, IL-17A induced expression of nicotinamide *N*-methyltransferase (*Nnmt*) in SAT and VAT of obese mice, but not of lean mice (Figure 3F). *Nnmt* gene-encoded protein catalyzes *N*-methylation of nicotinamide and other pyridines to form pyridinium ions. Nnmt knockdown has been shown to protect against diet-induced obesity [18]. IL-17A treatment increased expression of hormone-sensitive lipase (*Hsl*), uncoupling protein 1 (*Ucp1*), peroxisome proliferator-activated receptor-gamma coactivator 1α (*Pgc-1*α), and acyl-CoA oxidase 1, palmitoyl (*Acox1*) in SAT and VAT of both lean and obese mice (Figure 3G–J). *Hsl* gene-encoded lipase is capable of hydrolyzing a variety of esters. *Ucp1* gene-encoded mitochondrial protein separates oxidative phosphorylation from ATP synthesis with energy dissipated as heat. *Pgc-1*α gene-encoded protein regulates expression of multiple genes in energy metabolism. *Acox1* gene-encoded enzyme catalyzes fatty acid beta-oxidation. Two adipokine genes *leptin* and *adiponectin* were induced by IL-17A in SAT and VAT of both lean and obese mice, except *leptin* in lean SAT (Figure 3K,L).

### 2.4. IL-17A Differentially Activates Signaling Pathways in the Adipose Tissues of Lean and Obese Mice

**Figure 4:**
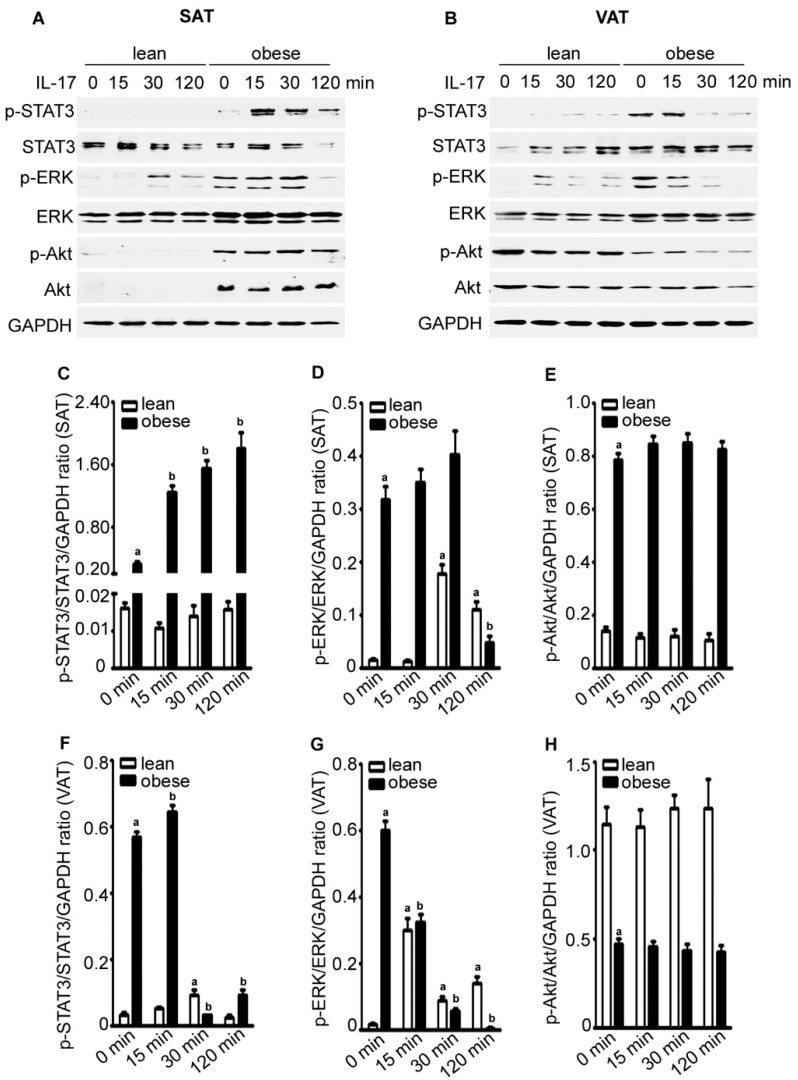
IL-17A differentially activates signaling pathways in the adipose tissues of lean and obese mice. (**A**,**B**) Adipose tissues were cultured in serum-free DMEM and treated without (time zero) or with 20 ng/mL IL-17A for 15, 30, and 120 min; proteins were assessed by Western blot analysis; GAPDH was probed for loading control; (**C**–**E**) Quantification of the Western blot results in (**A**) and (**F**–**H**) quantification of the Western blot results in (**B**); data are mean ± standard error of the mean (SEM) of three independent experiments; ^a^ *p* < 0.05, compared to the untreated group of the lean mice; ^b^ *p* < 0.05, compared to the untreated group of the obese mice.

We found that the basal levels of p-STAT3 (signal transducer and activator of transcription 3), p-ERK (extracellular signal-regulated kinase), and p-Akt were higher in obese SAT than lean SAT (Figure 4A,C–E). IL-17A treatment increased p-STAT3 levels in obese SAT, but not much in lean SAT. In contrast, IL-17A treatment increased p-ERK levels in lean SAT, but not much in obese SAT (and even reduced at 120 min) (Figure 4A,C,D). IL-17A did not have any effects on p-Akt levels in either lean or obese SAT (Figure 4A,E). We consistently found that the basal levels of p-STAT3 and p-ERK were higher in obese VAT than lean VAT, whereas the basal levels of p-Akt were lower in obese VAT than lean VAT (Figure 4B,F,H). IL-17A treatment slightly increased p-STAT3 levels in obese VAT and p-ERK levels in lean VAT, but did not have any effects on p-Akt levels (Figure 4B,F,H).

### 2.5. IL-17A Induces Adipokine Expression in Adipocytes

**Figure 5:**
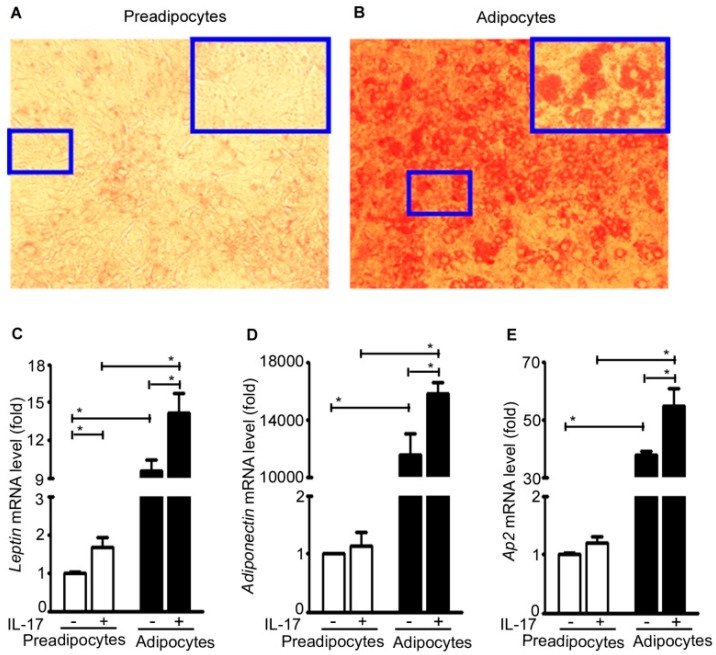
IL-17A induces adipokine expression in adipocytes. (**A**,**B**) Mouse 3T3-L1 pre-adipocytes were differentiated into mature adipocytes by 10-day differentiation culture; pictures are representatives of Oil Red O staining; original magnification, 100×, with the highlighted regions (small blue boxes) taken at 200× magnification (big blue boxes); (**C**–**E**) the pre-adipocytes and adipocytes were treated without (−) or with (+) 20 ng/mL IL-17A for 2 h; mRNA expression of each gene was analyzed with qRT-PCR; the pre-adipocytes without IL-17A treatment served as the basal level for comparison; data represent mean ± SEM (error bars) of six independent experiments (*n* = 6 per group), * *p* < 0.05 between the indicated groups.

Mouse 3T3-L1 preadipocytes were differentiated into mature adipocytes as evidenced by increased Oil Red O staining (Figure 5A,B). The basal level expression of *leptin*, *adiponectin*, and adipocyte protein *Ap2* (also named fatty acid binding protein 4, adipocyte, *Fabp4*) in the adipocytes was significantly increased compared to the preadipocytes (Figure 5C–E). Further, IL-17A treatment significantly increased expression of *leptin*, *adiponectin*, and *Ap2* in the adipocytes. In contrast, IL-17A significantly increased *leptin* expression, but not *adiponectin* or *Ap2* expression in the pre-adipocytes (Figure 5C–E).

### 2.6. IL-17A Differentially Induces Inflammatory Gene Expression in Pre-Adipocytes and Adipocytes

**Figure 6:**
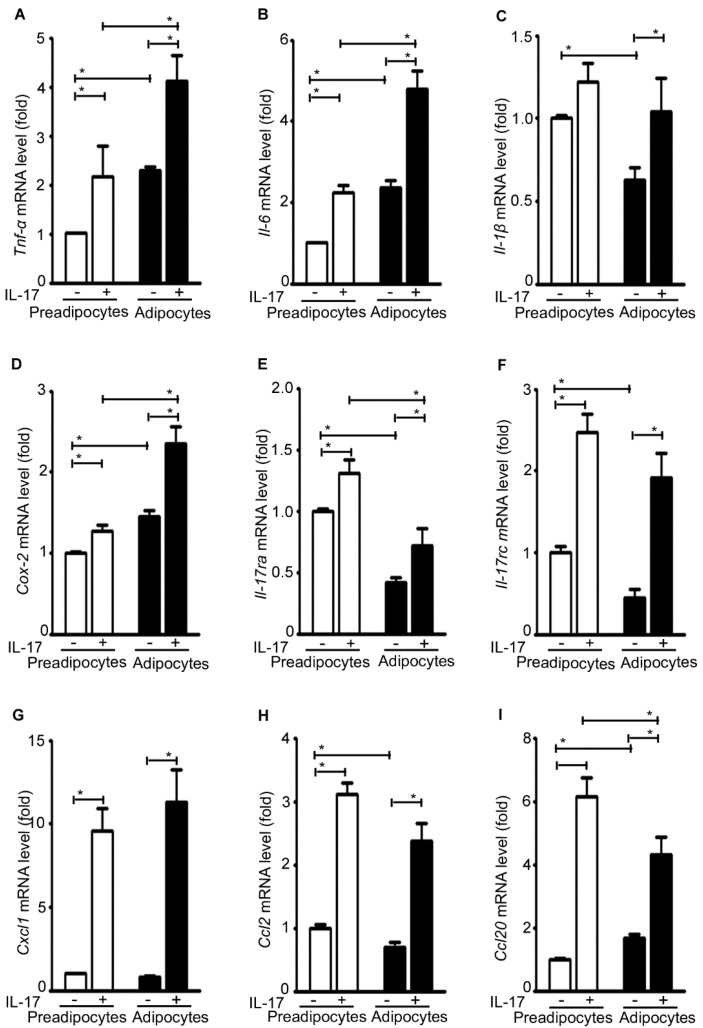
IL-17A differentially induces inflammatory gene expression in pre-adipocytes and adipocytes. (**A**–**I**) The pre-adipocytes and adipocytes were treated without (−) or with (+) 20 ng/mL IL-17A for 2 h; mRNA expression of each gene was analyzed with qRT-PCR; the pre-adipocytes without IL-17A treatment served as the basal level for comparison; data represent mean ± SEM (error bars) of six independent experiments (*n* = 6 per group), * *p* < 0.05 between the indicated groups.

The basal level expression of *Tnf-*α, *Il-6*, and *Cox-2* was significantly increased in the adipocytes compared to the pre-adipocytes, whereas the basal level expression of *Il-1*β, *IL-17ra*, and *IL-17rc* was significantly decreased in the adipocytes compared to the pre-adipocytes (Figure 6A–F). Nevertheless, IL-17A treatment significantly increased expression of *Tnf-*α, *Il-6*, *Il-1*β, *Cox-2*, *IL-17ra*, and *IL-17rc* in both the pre-adipocytes and adipocytes (Figure 6A–F). Similarly, IL-17A treatment significantly increased expression of *Cxcl1*, *Ccl2*, and *Ccl20* in both the pre-adipocytes and adipocytes (Figure 6G–I).

### 2.7. IL-17A Differentially Induces Metabolic Gene Expression in Pre-Adipocytes and Adipocytes

**Figure 7:**
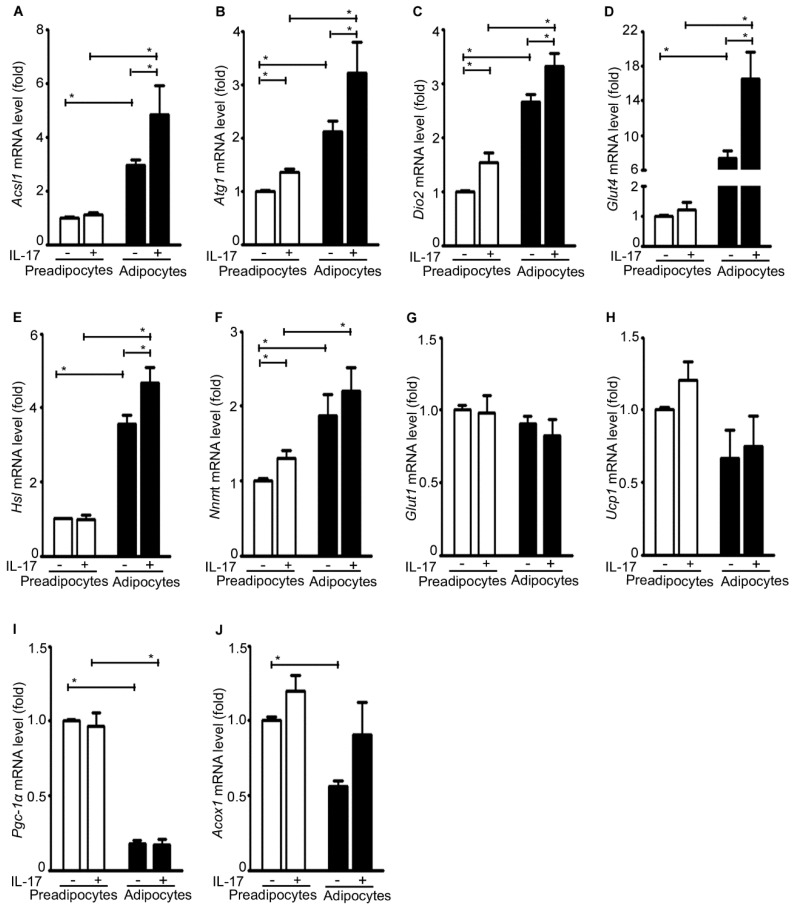
IL-17A differentially induces metabolic gene expression in pre-adipocytes and adipocytes. (**A**–**J**) The pre-adipocytes and adipocytes were treated without (−) or with (+) 20 ng/mL IL-17A for 2 h; mRNA expression of each gene was analyzed with qRT-PCR; the pre-adipocytes without IL-17A treatment served as the basal level for comparison; data represent mean ± SEM (error bars) of six independent experiments (*n* = 6 per group), * *p* < 0.05 between the indicated groups.

The basal level expression of *Acsl1*, *Atg1*, *Dio2*, *Glut4*, *Hsl*, and *Nnmt* was significantly higher in the adipocytes than the pre-adipocytes (Figure 7A–F). In contrast, the basal level expression of *Ucp1*, *Pgc-1*α, and *Acox1* was significantly lower in the adipocytes than the pre-adipocytes (Figure 7H–J). There was no difference in the basal level expression of *Glut1* between the adipocytes and the pre-adipocytes (Figure 7G). IL-17A treatment significantly increased expression of *Acsl1*, *Atg1*, *Dio2*, *Glut4*, and *Hsl* in the adipocytes (Figure 7A–E), and also increased expression of *Atg1*, *Dio2*, and *Nnmt* in the pre-adipocytes (Figure 7B,C,F). In contrast, IL-17A did not have any significant effects on expression of *Glut1*, *Ucp1*, *Pgc-1*α, and *Acox1* (Figure 7G–J).

### 2.8. IL-17A Differentially Activates Signaling Pathways in Preadipocytes and Adipocytes

**Figure 8:**
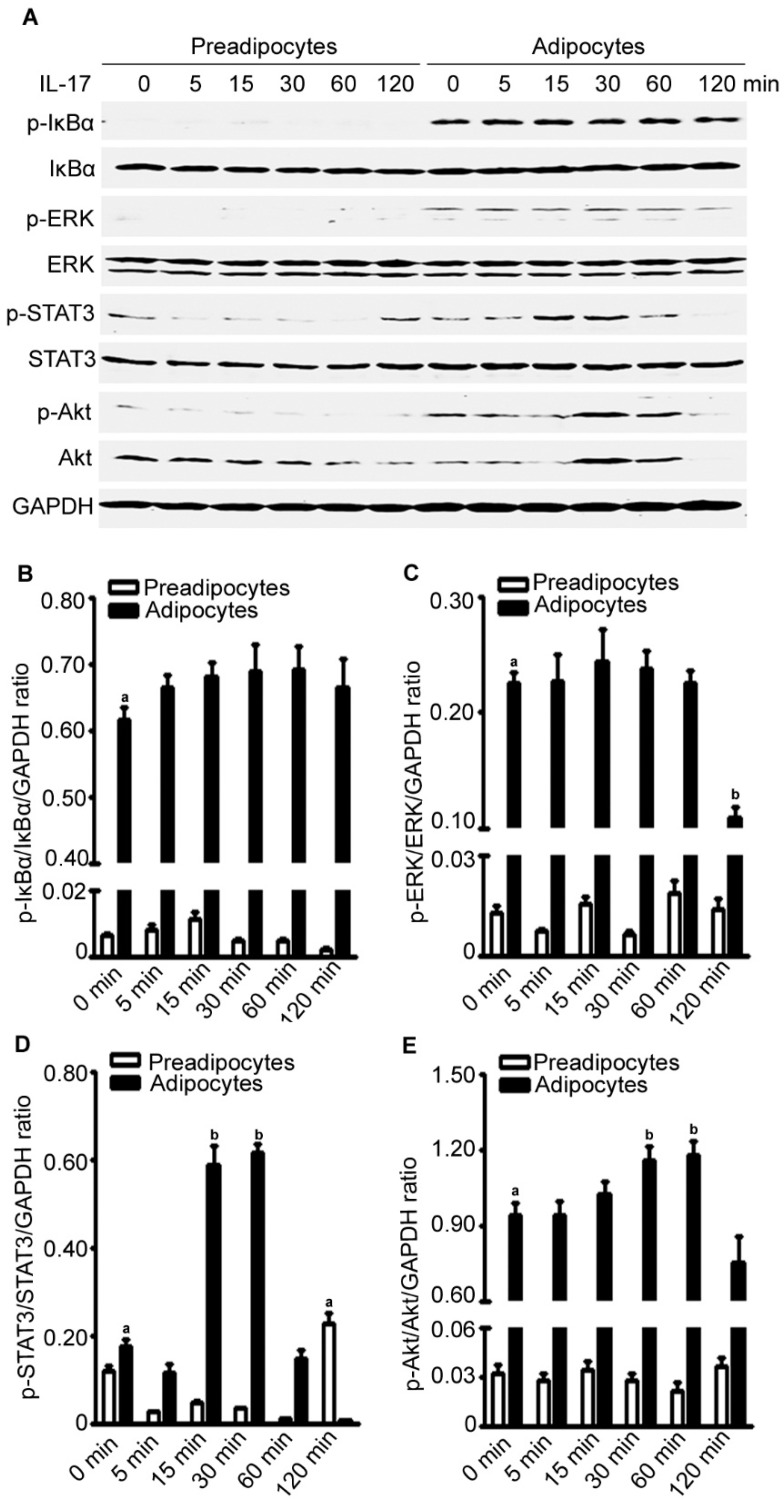
IL-17A differentially activates signaling pathways in pre-adipocytes and adipocytes. (**A**) The pre-adipocytes and adipocytes were treated without (time zero) or with 20 ng/mL IL-17A for 5, 15, 30, 60, and 120 min; proteins were assessed by Western blot analysis; GAPDH was probed for loading control; (**B**–**E**) quantification of the Western blot results in (**A**); data are mean ± standard error of the mean (SEM) of three independent experiments; ^a^ *p* < 0.05, compared to the untreated group of the pre-adipocytes; ^b^ *p* < 0.05, compared to the untreated group of the adipocytes.

We found that the basal levels of p-IκBα, p-ERK, and p-Akt were higher in the adipocytes than the pre-adipocytes (Figure 8A–E). In the adipocytes, IL-17A treatment slighted increased p-IκBα levels at 5 to 120 min after the treatment, while it dramatically increased p-STAT3 levels at 15 to 30 min and p-Akt levels at 30 to 60 min after treatment (Figure 8A–E). In the pre-adipocytes, IL-17A treatment only slightly increased p-STAT3 levels at 120 min after treatment (Figure 8A–E). The time-dependent differences in IL-17A-activated signaling pathways between pre-adipocytes and adipocytes are shown in Figure 8B–E.

## 3. Discussion

Obesity and obesity-associated comorbidities are tightly linked to inflammation [8]. VAT and SAT, particularly VAT, have been found to be inflammatory depots in humans [19]. Diet-induced obesity increases the Th17 cell number in the mouse spleen, which predisposes the animals to autoimmune diseases [9]. Diet-induced obesity also increases Th17 cell number in mouse adipose tissue because adipose tissue dendritic cells secrete higher levels of IL-6, TGF-β, and IL-23 to promote Th17 cell generation [20]. Our study found that obese SAT had higher levels of IL-17A protein than lean SAT, which is consistent to the previous report [20].

Given that IL-17A is expressed at high levels in adipose tissue, we decided to assess IL-17A’s functions in adipose tissues of lean and obese mice. We found that the basal levels of *Tnf-*α, *Il-6*, and *Il-1*β were higher in VAT than SAT in both lean and obese mice, and the basal levels of *Tnf-*α, *Il-6*, and *Il-1*β were higher in the obese adipose tissues than lean adipose tissues. These findings were consistent with the previous results in humans [19]. We found that IL-17A treatment increased expression of *Tnf-*α, *Il-6*, and *Il-1*β in SAT and VAT with a higher amplitude in obese adipose tissues than lean adipose tissues. IL-17A treatment also increased expression of *Cox-2*, *IL-17ra*, *IL-17rc*, *Cxcl1*, and *Ccl2*, albeit at different amplitudes. In addition, we found that IL-17A treatment increased expression of multiple metabolic genes, such as *Acsl1*, *Atg1*, *Dio2*, *Glut4*, *Nnmt*, *Hsl*, *Ucp1*, *Pgc-1*α, and *Acox1*. These findings suggest that IL-17A may affect metabolism in adipose tissues. TNF-α, IL-6, and IL-1β have been known for their roles in impairing insulin signaling [7]. Further studies are warranted to determine whether and how IL-17A is associated with metabolic dysfunctions.

Since adipose tissue contains a variety of cells including pre-adipocytes, adipocytes, fibroblasts, endothelial cells, and immune cells, we did not know how much effects of IL-17A were on the adipocytes. Therefore, we used mouse 3T3-L1 pre-adipocyte-derived adipocytes to investigate IL-17A’s effects on the adipocytes. We found that IL-17A induced expression of inflammatory genes, such as *Tnf-*α, *Il-6*, *Il-1*β, *Cox-2*, *IL-17ra*, *IL-17rc*, *Cxcl1*, *Ccl2*, and *Ccl20* in both pre-adipocytes and adipocytes. IL-17A also induced expression of metabolic genes, such as *Acsl1*, *Atg1*, *Dio2*, *Glut4*, and *Hsl1* in the adipocytes. However, IL-17A induced expression of only *Atg1*, *Dio2*, and *Nnmt* in the pre-adipocytes. In general, the effects of IL-17A on adipocytes appear consistent with its effects on adipose tissues. Nevertheless, there are some differences between adipocytes and adipose tissues in regard to the specific genes and amplitudes of induction by IL-17A. We believe that the differences are due to the heterogeneity of the cells in adipose tissues. We did notice that the basal levels of activated signaling pathways such as ERK and Akt were increased in both obese adipose tissues and adipocytes, and IL-17A treatment further enhanced activation of STAT3 in both obese adipose tissues and adipocytes. IL-17A activates the signaling pathways and induces gene expression differently between the adipose tissues and adipocytes may suggest a tissue-specific function of IL-17A. This is further suggested by an early study showing a different gene expression profile induced by IL-17A in the pre-osteoblast cell line MC3T3-E1 [21]. Our findings indicate that IL-17A differentially induces inflammatory and metabolic gene expression in the adipose tissues of lean and obese mice.

## 4. Materials and Methods

### 4.1. Animals and Adipose Tissue Culture

Animal study was approved by the Animal Care and Use Committee of Tulane University (Protocol# 4125R2, 3/19/2015–3/18/2018). Twelve male mice (strain genetic background C.129S4 (B6)) were used. At three weeks of age, six mice (*n* = 6) were fed with a regular diet (13.2% calories by fat, cat# 5053, LabDiet, Brentwood, MO, USA) and six mice (*n* = 6) were fed with a high-fat diet (60% calories by fat, cat# D12492, Research Diets, New Brunswick, NJ, USA). The diet compositions provided by the manufacturers are shown in Table 1 and Table 2. Animal body weight was measured weekly. At 30 weeks of age, animals were euthanized. Subcutaneous adipose tissue (SAT) was dissected from bilateral inguinal regions. Visceral adipose tissue (VAT) was dissected from bilateral epididymal adipose tissue pads. After weighing, a portion of SAT and VAT was homogenized for isolating proteins for enzyme-linked immunosorbent assay (ELISA) of IL-17A. The remaining SAT and VAT were washed three times with phosphate-buffered saline (PBS, pH 7.40) and cut into 1–2 mm^3^ cubes. A portion of adipose tissue cubes was randomly split into a control group and a treatment group cultured in serum-free Dulbecco’s Modified Eagle Medium (DMEM, pH 7.40, Mediatech, Inc., Manassas, VA, USA) in 1.5-mL Eppendorf tubes in a 5% CO_2_ humidified incubator at 37 °C. The control group was treated with PBS and the treatment group was treated with 20 ng/mL recombinant mouse IL-17A (R and D Systems, Inc., Minneapolis, MN, USA) for 2 h, in order to assess the direct effects of IL-17A. Then, the adipose tissues were homogenized for RNA isolation. Similarly, another portion of adipose tissue cubes was randomly split into a control group and a treatment group cultured in serum-free DMEM and treated with PBS (control group) or 20 ng/mL IL-17A for 15, 30, and 120 min. After treatment, the adipose tissues were homogenized for isolating proteins for Western blot analysis.

### 4.2. Cell Culture and Adipocyte Differentiation

Mouse 3T3-L1 cell line was obtained from American Type Culture Collection (ATCC, Manassas, VA, USA), which is considered as a pre-adipocyte cell line that may differentiate into mature adipocytes in a confluent and contact inhibited state [22]. 6 × 10^5^ cells/well were seeded into six-well plates and incubated in DMEM supplemented with 10% bovine calf serum (BCS; Mediatech, Inc.) and 1% penicillin/streptomycin in a 5% CO_2_ humidified incubator at 37 °C. Two days later, the cells grew into full confluence and were cultured in differentiation medium containing DMEM supplemented with 10% fetal bovine serum (FBS), 10 μg/mL insulin, 1 μM dexamethasone, 0.5 mM 3-isobutyl-1-methylxanthine, and 2 μM rosiglitazone (Sigma-Aldrich, St. Louis, MO, USA) [23,24,25]. After two days, the medium was changed to DMEM with 10% FBS and 10 μg/mL insulin. Two days later, the medium was changed to DMEM with 10% FBS only every two days. After a total of 10 days of adipocyte differentiation, representative wells were randomly selected, fixed with 10% formalin, and stained with Oil Red O (Sigma-Aldrich) for 10 min at room temperature to assess lipid accumulation in mature adipocytes. The pre-adipocytes and mature adipocytes were cultured in serum-free DMEM for 12 h and then treated with PBS (control group) or 20 ng/mL IL-17A for 2 h followed by RNA isolation, or treated for 5, 15, 30, 60, and 120 min followed by protein isolation and Western blot analysis.

### 4.3. Quantitative Real-Time Reverse Transcriptase Polymerase Chain Reaction (qRT-PCR)

Total RNAs were isolated from homogenized adipose tissues or cells using RNeasy Kit (QIAGEN, Valencia, CA, USA) according to the manufacturer’s instructions. Genomic DNA contamination was avoided by using DNaseI digestion. RNA was reversed to cDNA using iScript™ cDNA synthesis kit (Bio-Rad Laboratories, Hercules, CA, USA). All PCR primers were obtained from Eurofins (Huntsville, AL, USA) and the sequences are shown in Table 3. Real-time PCR was conducted using iQ5^®^ iCycler and iQ™SYBRGreen Supermix (Bio-Rad Laboratories) following the manufacturer’s instructions. The result of each group was normalized to its own glyceraldehyde-3-phosphate dehydrogenase (*Gapdh*) level using the formula Δ*C*_t_ (Cycle threshold) = *C*_t_ of target gene—*C*_t_ of *Gapdh*. The fold change of the mRNA level of each treatment group was calculated as 2^−ΔΔ*C*t^, where ΔΔ*C*_t_ = Δ*C*_t_ of the target gene in the treatment group—Δ*C*_t_ of the target gene in the control group.

### 4.4. Western Blot Analysis

Proteins were extracted from homogenized adipose tissues or cells in the lysis buffer containing 50 mM sodium fluoride, 0.5% Igepal CA-630 (NP-40), 10 mM sodium phosphate, 150 mM sodium chloride, 25 mM Tris (pH 8.0), 1 mM phenylmethylsulfonyl fluoride, 2 mM ethylenediaminetetraacetic acid (EDTA), and 1.2 mM sodium vanadate. The protein concentration was measured using Bio-Rad protein Assay Dye Reagent Concentrate (Bio-Rad Laboratories, Hercules, CA, USA) and BioTek ELx800 microplate reader (BioTek, Winooski, VT, USA). Approximately 80 μg of protein was run on 10% sodium dodecyl sulfate-polyacrylamide gel electrophoresis and transferred to polyvinylidene difluoride membrane. 5% non-fat dry milk in TBST buffer (25 mM Tris-HCl, 125 mM sodium chloride and 0.1% Tween 20) was used to block the membrane. The membrane was incubated with primary antibodies at 4 °C overnight and then incubated with IRDye^®^ 800CW- or IRDye^®^ 680RD-conjugated secondary antibodies (LI-COR Biosciences, Lincoln, NE, USA) at room temperature for one hour. The results were scanned with an Odyssey Infrared Imager (LI-COR Biosciences). For loading control, the membrane was re-probed for GAPDH. Quantification of the Western blot signals was performed using ImageJ system (an image analysis software developed by the National Institutes of Health of the United States of America). The integrated density values of each phosphorylated protein were normalized first by the corresponding unphosphorylated protein level and then by the corresponding GAPDH level. The results represent mean ± standard error of the mean (SEM) of three independent experiments. The antibodies used were: rabbit anti-p-Akt (S473), rabbit anti-Akt, rabbit anti-p-STAT3, mouse anti-STAT3, rabbit anti-p-IκBα, and mouse anti-IκBα antibodies that were purchased from Cell Signaling Technology, Danvers, MA, USA; mouse anti-p-ERK1/2(E-4) and rabbit anti-ERK1/2 antibodies that were purchased from Santa Cruz Biotechnology, Dallas, TX, USA; and mouse anti-GAPDH antibody that was purchased from Millipore, Billerica, MA, USA.

### 4.5. ELISA Assay

The IL-17A protein levels in mouse adipose tissues were measured using an ELISA kit (RayBiotech, Inc., Norcross, GA, USA) according to the manufacturer’s instructions. Each protein sample from adipose tissue homogenization was quantified for protein concentration and then was analyzed in duplicate wells in 96-well plates. The mean of the duplicate readings was used to calculate IL-17A concentrations (unit: pg/mL) using a linear regression curve derived from a series of IL-17A protein standards provided in the kit. Then, IL-17A levels (pg/mg tissue protein) in the adipose tissues were calculated as IL-17A concentration (unit: pg/mL) divided by the tissue protein concentration (unit: mg/mL) of each protein sample.

### 4.6. Statistical Analysis

Statistical analysis was performed using GraphPad Prism software version 6.1 (GraphPad Software, La Jolla, CA, USA). The results were presented as mean ± standard error of the mean (SEM) and were analyzed using Student’s *t*-test and two-way analysis of variance (ANOVA) and Tukey’s test. *p* < 0.05 was considered as statistical significant.

## 5. Conclusions

The present study demonstrates that IL-17A levels are higher in obese SAT than lean SAT. The basal level expression of inflammatory and metabolic genes is different between SAT and VAT and between lean and obese adipose tissues. IL-17A differentially induces inflammatory and metabolic gene expression in the adipose tissues of lean and obese mice. These findings suggest that IL-17A may play important roles in obesity-associated inflammation and metabolic disorders.

## Figures and Tables

**Table 1:** Diet composition of the regular diet (cat# 5053, LabDiet, Brentwood, MO, USA).

Nutrients				Minerals		Vitamins	
Protein, %	21.0	Fat (ether extract), %	5.0	Ash, %	5.9	Carotene, ppm	1.5
Arginine, %	1.28	Fat (acid hydrolysis), %	6.3	Calcium, %	0.81	Vitamin K, ppm	3.3
Cystine, %	0.36	Cholesterol, ppm	142	Phosphorus, %	0.65	Thiamin hydrochloride, ppm	17
Glycine, %	0.96	Linoleic acid, %	2.14	Phosphorus (non-phytate), %	0.34	Riboflavin, ppm	8.0
Histidine, %	0.52	Linolenic acid, %	0.27	Potassium, %	1.09	Niacin, ppm	85
Isoleucine, %	0.86	Arachidonic acid, %	<0.01	Magnessium, %	0.23	Pantothenic acid, ppm	17
Leucine, %	1.56	Omega-3 fatty acids, %	0.44	Sulfur, %	0.33	Choline chloride, ppm	2000
Lysine, %	1.17	Total saturated fatty acids, %	0.78	Sodium, %	0.30	Folic acid, ppm	3.0
Methionine, %	0.62	Total monounsaturated fatty acids, %	0.97	Chloride, %	0.52	Pyridoxine, ppm	9.6
Phenylalanine, %	0.91	Fiber (crude), %	4.5	Fluorine, ppm	9.3	Biotin, ppm	0.30
Tyrosine, %	0.59	Neutral detergent fiber, %	15.8	Iron, ppm	200	B12, mcg/kg	51
Threonine, %	0.78	Acid detergent fiber, %	5.8	Zinc, ppm	89	Vitamin A, IU/gm	15
Tryptophan, %	0.24	Nitrogen-free extract, %	53.5	Manganese, ppm	84	Vitamin D3, IU/gm	2.3
Valine, %	0.96	Starch, %	28.6	Copper, ppm	14	Vitamin E, IU/kg	99
Serine, %	0.98	Glucose, %	0.19	Cobalt, ppm	0.71	-	-
Aspartic acid, %	2.18	Fructose, %	0.24	Iodine, ppm	0.97	Calories provided by	
Glutamic acid, %	4.16	Sucrose, %	3.24	Chromium, ppm	0.01	Protein, %	24.496
Alanine, %	1.19	Lactose, %	1.34	Selenium, ppm	0.37	Fat, %	13.123
Proline, %	1.31	Total digestible nutrients, %	75.1	-	-	Carbohydrates, %	62.380
Taurine, %	0.03	-	-	-	-	-	-

**Table 2:** Diet Composition of the high-fat diet (cat# D12492, Research Diets, New Brunswick, NJ, USA).

Composition	Gram (gm)%	Kcal %
Protein	26.2	20
Carbohydrate	26.3	20
Fat	34.9	60
Total	-	100
kcal/gm	5.24	-
Ingredient	gm	kcal
Casein, 30 mesh	200	800
l-Cysteine	3	12
Corn starch	0	0
Maltodextrin 10	125	500
Sucrose	68.8	275.2
Cellulose, BW200	50	0
Soybean oil	25	225
Lard	245	2205
Mineral mix S10026	10	0
Dicalcium phosphate	13	0
Potassium citrate, 1 H_2_O	16.5	0
Vitamin mix V10001	10	40
Choline bitartrate	2	0
Brillant Blue For Coloring Food (FD & C Blue Dye # 1)	0.05	0
Total	773.85	4057

**Table 3:** PCR primer sequences.

Gene	Forward (5′ to 3′)	Reverse (5′ to 3′)
*Tnf-a*	CTACTCCCAGGTTCTCTTCAA	GCAGAGAGGAGGTTGACTTTC
*Il-6*	CCACTTCACAAGTCGGAGGCTTA	GCAAGTGCATCATCGTTGTTCATAC
*Il-1*β	TCTTCTTTGGGTATTGCTTGG	TGTAATGAAGACGGCACACC
Cox-2	TGGAAAAGGTTCTTCTACGGAG	TGAACCCAGGTCCTCGCT
*Il-17ra*	CGGAGAATTAGTCCCTGTGTTG	GAACAGTCACTTCATACTCCTGG
*Il-17rc*	TTCTGCGGTATTTGACTGTTTCG	GTCCCGGACTTCAAGACCC
*Cxcl1*	CACCCAAACCGAAGTCATAG	AAGCCAGCGTTCACCAGA
*Ccl2*	GCCTGCTGTTCACAGTTGC	TGTATGTCTGGACCCATTCCT
*Ccl20*	GTCCAATTCCATCCCAAAAA	AACTGGGTGAAAAGGGCTGT
*Acsl1*	GATCTGGTGGAACGAGGCAA	CTTCGGGTTCTGGAGGCTTG
*Atgl*	CAGCACATTTATCCCGGTGTAC	AAATGCCGCCATCCACATAG
*Dio2*	GTCCGCAAATGACCCCTTT	CCCACCCACTCTCTGACTTTC
*Glut1*	TCAACACGGCCTTCACTG	CACGATGCTCAGATAGGACATC
*Glut4*	TTGGCTCCCTTCAGTTTGG	CTACCCAGCCACGTTGCAT
*Nnmt*	AGGAACCAGGAGCCTTTGACT	CCTGAGGGCAGTGCGATAGG
*Ucp1*	GGCTGGTGGTGGTCGGAGAT	CCGAAGGCAGAAGTGAAGTG
*Pgc1a*	AGACAAATGTGCTTCGAAAAAGAA	GAAGAGATAAAGTTGTTGGTTTGGC
*Acox1*	GCCCAACTGTGACTTCCATTAA	GTAGCACTCCCCTCGAGTGAT
*Leptin*	CTGCCCCCCAGTTTGATG	GCCAGGCTGCCAGAATTG
*Adiponectin*	AACTTGTGCAGGTTGGATGG	GCCCTTCAGCTCCTGTCATT
*Ap2*	ACACCGAGATTTCCTTCA AACTG	CCATCTAGGGTTATG TGCTCTTCA
*Gapdh*	TGCACCACCAACTGCTTAG	GGATGCAGGGATGATGTTC
